# Acute chemoradiotherapy toxicity in cervical cancer patients

**DOI:** 10.1515/med-2020-0222

**Published:** 2020-09-02

**Authors:** Marija Zivkovic Radojevic, Aleksandar Tomasevic, Vesna Plesinac Karapandzic, Neda Milosavljevic, Slobodan Jankovic, Marko Folic

**Affiliations:** Centre for Oncology and Radiology, Clinical Centre Kragujevac, Zmaj Jovina Street 30, 34000, Kragujevac, Serbia; University of Kragujevac, Faculty of Medical Sciences, Kragujevac, Serbia; Institute for Oncology and Radiology, Brachytherapy Department, Belgrade, Serbia; University of Belgrade, Faculty of Medicine, Belgrade, Serbia; Clinical Centre Kragujevac, Clinical Pharmacology Department, Kragujevac, Serbia

**Keywords:** cervical cancer, radiotherapy, acute radiation toxicity, ACE inhibitors

## Abstract

During radiotherapy treatment for cervical cancer, up to 84% of patients exhibit some form of acute radiation toxicity (ART). The primary aim of this clinical study is to determine the impact of angiotensin-converting enzyme (ACE) inhibitors, β-blockers and other risk factors such as the patient’s anatomical characteristics on ART emergence in patients with locally advanced cervical cancer treated by chemoradiotherapy. This is a combination of two nested case–control studies within the cohort of patients with locally advanced cervical cancer based on the analysis of potential risk factors for the onset of ART in patients treated with 3D conformal radiotherapy (3D-CRT) and 2D conventional radiotherapy (2D-RT), prospectively followed up from January 2017 to September 2018 in a tertiary care hospital. The ACE inhibitors and bladder volume were identified as factors that significantly affect the occurrence of ART in patients treated with 3D-CRT. In patients treated with 2D-RT, the factors that significantly affect the occurrence of ART were ACE inhibitors, body mass index (BMI), brachytherapy rectal and bladder dose. This study has shown that BMI, radiation dose received by the bladder and rectum are of exceptional importance for the occurrence of the ART and also that therapy with ACE inhibitors was associated with the decreased chances of the ART.

## 1 Introduction

Cervical cancer is the fourth most common malignancy among women [1]. The gold-standard treatment for locally advanced cervical cancer (Federation Internationale de Gynecologie etd’ Obstetrique (FIGO) stage IIb to IVa) is concurrent chemoradiotherapy (CCRT), including chemotherapy with external beam radiotherapy (EBRT), followed by brachytherapy [2,3].

Radiotherapy treatment can cause acute radiation toxicity (ART) (within 90 days, from initiation of treatment) and/or chronic radiation toxicity (months and years after completion of radiotherapy). During radiotherapy treatment for cervical cancer, up to 84% of patients exhibit some form of ART [4,5,6]. The most common manifestations are hematological, gastrointestinal or genitourinary toxicity. The intensity and severity of these adverse radiotherapy effects depend on the radiation dose, the fractionation regime, the radiation technique applied and the duration of the treatment [6]. However, the interactions among the patient’s individual characteristics, the disease stage, genetic aspects, comorbidities and other applied therapeutic modalities are also known to be factors [7,8,9,10]. The emergence of serious ART is one of the most important causes of the development of chronic toxicity, which often requires extensive intervention and increasing costs of the long-term treatment. As a result, there is ample incentive to work on the timely and accurate identification and monitoring of patients at an increased risk of developing ART.

Also, it is unknown whether the individual therapy, prescribed for the treatment of other nononcological diseases, during radiation can contribute to the emergence and severity of ART. Given that antihypertensives are among the most prescribed drugs, and that their action is manifested through receptors expressed in various tissues and organs, their use might exert certain influence on the effects of the radiotherapy applied. Earlier experimental studies have shown that angiotensin-converting enzyme (ACE) inhibitors have some effect on reducing radiation toxicity, while the influence of one of the most commonly used drugs in light of ART during the radiotherapy remains controversial [11,12,13,14,15,16,17,18,19].

The primary aim of this clinical study is to determine the impact of ACE inhibitors, β-blockers and other potential risk factors such as the patient’s pelvic and anatomical characteristics on ART emergence in patients with locally advanced cervical cancer treated by chemoradiotherapy.

## 2 Subjects and methods

### 2.1 Study design

This study was designed as the combination of two nested case–control studies within the cohort of patients with locally advanced cervical cancer based on the analysis of potential risk factors important for the onset of ART in 54 patients treated with 3D conformal radiotherapy (3D-CRT) and 84 patients treated with 2D conventional radiotherapy (2D-RT), prospectively followed up from January 2017 to September 2018 in a tertiary care hospital. This study was reviewed and approved by the Ethics Committee.

### 2.2 Study sample

All patients who met the inclusion criteria for study participation were monitored during the CCRT, following the informed consent. Depending on the outcome of the occurrence of ART underway 2D-RT or 3D-CRT, they were classified into two groups. The group of cases consisted of patients with verified locally advanced cervical cancer, initially treated with CCRT, who developed ART grade 2 or higher according to the relevant Common Terminology Criteria for Adverse Events version 4.03 during radiotherapy treatment [20]. The number of case was equal to the number of control besides the fact that the gender of the patients is the same and age of patients was a matching criterion. For each case, all possible match controls were found from the cohort group. If there were multiple match controls for one case, only one was chosen by randomization using a random number generator from SPSS-18 statistical software.

All patients, without exception, were subjected to standard preventative measures for ART minimization according to the hospital protocol. These measures include the prevention of gastrointestinal toxicity (prescribing a hygienic diet regimen during radiotherapy), hematology (control of laboratory analyzes once a week) and genitourinary toxicities (maintaining hygiene, adequate fluid intake, urine culture, providing protocols to ensure constant bladder overload) as well as adherence to radiotherapy constrains for the organs at risk.

The inclusion criteria were as follows: 18–80 years of age, pathohistologically verified cervical cancer, FIGO stages from IIb to IVa, good general condition estimated based on the reference scale of the Eastern Cooperative Oncology Group or performance status 0 to 2 [21] and the completed CCRT treatment. Criteria for exclusion from the research were the presence of mental illness, pregnancy and lactation.

Standard CCRT treatment included:

(1) Cisplatin-based chemopotentiation: at a dose of 40 mg/m^2^ calculated body surface area using Mosteller’s formula, administered 2 hours before radiotherapy course, once a week.
(2) Radiotherapy (EBRT + brachytherapy).

The study included two cohorts of patients treated in identical manner according to the hospital protocol, the only difference being different radiotherapy techniques used to adjust for the effect of a radiotherapy technique on the occurrence of acute radiation toxicity manifestations.

### 2.3 2D-RT

EBRT planning was conducted on an X-ray simulator. Radiotherapy was performed on a linear accelerator, in a 5 day regimen, with parallel opposite fields (2D technique) and photons of energy from 6 to 10 MV. A EBRT dose from 45 to 50.4 Gy was applied using a standard fractionation regimen (1.8 Gy per day). In patients with verified paraaortic lymphadenopathy involvement, expanded fields were used [22].

### 2.4 3D-CRT

3D-CRT planning was conducted on a computer tomography (CT) simulator. The same fractionation and dosing regimen were used as for the patients treated with 2D-RT with four fields (box technique). Target volume delineation and organ at risk were contoured according to the Radiation Therapy Oncology Group [23].

### 2.5 Brachytherapy

High-dose rate (HDR) brachytherapy was performed using a remote afterloading technique. All patients were treated with a dose of 6–7 Gy per fraction in four to five sessions [24]. The dose at point A and point B was calculated based on the recommendations of the International Commission on Radiation Units (ICRU Report No. 38) [25]. Rectal and bladder doses were calculated to the ICRU points using two orthogonal radiographies with the contrast placed in these organs at risk.

### 2.6 Personal therapy, patients and treatment characteristics

Prior to commencement of cancer treatment, patients were receiving their usual therapeutic doses of ACE inhibitors and β-blockers as prescribed by a general practitioner or cardiologist and in accordance with therapeutic indications. The use of the following ACE inhibitors was recorded: enalapril (10–20 mg daily), perindopril (4–8 mg daily), ramipril (5–10 mg daily), fosinopril (10–20 mg daily). Patients were receiving selective and non-selective beta blockers – propranolol (40–160 mg), atenolol (100 mg), and bisoprolol (5 mg).

The following variables were examined: sociodemographic data (age, education, Charlson’s comorbidity–age combined risk score [26] and presence of hypertension); patients habits (cigarette smoking and alcohol consumption); data from the medical records (previous abdominal or pelvic surgery, number of years since intervention, pathohistological type of tumor, FIGO stage, administration of ACE inhibitors, β-blockers and over four cycles of chemotherapy), parameters related to radiotherapy technique (surface of radiation fields, paraaortic fields, photon energy, uterine probe over 5 cm, bladder and rectal doses and duration of radiotherapy treatment), adherence to the prescribed diet regime, relevant measurements of interest in terms of body height and weight, waist circumference and bi-spinal diameter (with the help of a Breisky pelvimeter). Based on available parameters, pelvic gross volume was obtained using the formula for calculating the volume of a sealed coupe:

}{}V=\frac{H\text{π}}{3}({R}^{2}+Rr+{r}^{2})

where *H* is radiotherapy fields height, *R* is bi-spinal diameter and *r* is radiotherapy field width.

In contrast to the patients who received 2D-RT, in the patients treated with 3D-CRT, we measured the pelvic gross volume, subcutaneous fatty tissue and the volumes of organs at risk (bladder, rectal and bowel bag) using a CT-based three-dimensional approach. The pelvic volume was determined by contouring the volume beginning from the front of the pelvic spines, back to the L4–L5 vertebrae level and then to the vaginal introitus.

Although all examined variables were monitored and recorded weekly, the values for analyzed clinical variables presented in the Section 3 were measured immediately prior to initiation of therapy.

### 2.7 Statistics

The collected data were processed using descriptive statistics. For continuous variables, the significance of the difference was tested using the parametric Student’s *t*-test and nonparametric tests (Mann–Whitney *U* test) in case of nonnormal data distribution. The *χ* ^2^ test was used for categorical variables. The differences in the compared data were considered statistically significant if the probability of the null hypothesis was less than 5% (*p* < 0.05). The variables which turned out to be significant predictors for ART after univariate logistic analysis were then put through a multivariate binary logistic regression. Benjamini–Hochberg method was used to control the false discovery rate for multicomparison *p*-value correction. SPSS-18 statistical software for Windows was used to calculate and process the data.

## 3 Results

Fifty-four patients treated with 3D-CRT and 84 patients treated with 2D-RT who had given their informed consent and had completed the entire CCRT treatment were included in this study.

### 3.1 3D-CRT

The main characteristics of the patients treated with 3D-CRT and the differences between groups are presented in Table 1. Univariate analysis showed that cigarette smoking had a statistically significant impact on the onset ART. The analysis of the specific drugs used during radiotherapy, the chemotherapy regime, showed that ACE inhibitor therapy, concurrent with radiotherapy, had a statistically significant effect on the appearance of ART. The parameters related to the applied radiotherapy technique, which proved to be statistically significant, were a pause in therapy of more than 7 days and bladder volume. In addition to the constitutional parameters that proved to be statistically significant, we assessed the possible impact on the appearance of ART of weight ≤ 60 kg, BMI under 21 kg/m^2^, gross pelvic volume and volume of the pelvic subcutaneous fatty tissue.

**Table 1:** Analysis of potential risk factors important for ART onset in patient treated with 3D-CRT

Variables	Univariate analysis			Multivariate analysis	
	With toxicity (*n* = 27)	Without toxicity (*n* = 27)	Test and *p*-values	Exp(B) (95% CI for ExpB)	*p*
Age	50.41 (±9.162)	49.78 (±9.078)	*t* = −254		
			*p* = 0.801^a^		
Education groups of patients (years)					
Primary school	3 (11.1%)	1 (3.7%)	*χ* ^2^ = 1.730		
high school	16 (59.3%)	20 (74.1%)	*p* = 0.421		
College	8 (26.6%)	6 (22.2%)			
Charlson's comorbidity-age combined risk score					
Score 2 (mildly ill)	5 (18.5%)	5 (18.5%)	*χ* ^2^ = 0.929		
Score 3 (moderately ill)	5 (18.5%)	7 (25.9%)	*p* = 0.818		
Score 4 (severely ill)	7 (25.9%)	8 (29.6%)			
Score 5 (severely ill)	10 (37.0%)	7 (25.9%)			
Hypertension	8 (44.4%)	10 (37.0%)	*χ* ^2^ = 0.247		
			*p* = 0.619		
Smoking	16 (59.3%)	8 (29.6%)	*χ* ^2^ = 4.800	1.043 (0.109–9.991)	*p* = 0.971
			*p* = 0.028		
Alcohol	4 (14.8%)	3 (11.1%)	*χ* ^2^ = 0.164		
			*p* = 0.685		
Surgery in the abdomen or small pelvis	10 (37.0%)	9 (33.3%)	*χ* ^2^ = 0.081		
			*p* = 0.776		
Patohistological type of tumor					
Squamocellular	26 (96.3%)	24 (88.9%)	*χ* ^2^ = 1.080		
Adenocarcinoma	3 (11.1%)	1 (3.7%)	*p* = 0.299		
FIGO stage					
IIb	9 (33.3%)	7 (25.9%)	*χ* ^2^ = 1.972		
IIIa	7 (25.9%)	10 (37.0%)	*p* = 0.578		
IIIb	8 (29.6%)	5 (18.5%)			
IVa	3 (11.1%)	5 (18.5%)			
ACE inhibitors	15 (55.6%)	7 (25.9%)	*χ* ^2^ = 4.909	0.060 (0.004–0.817)	*p* = 0.035
			*p* = 0.027		
β-Blockers	4 (14.8%)	10 (37.0%)	*χ* ^2^ = 3.471		
			*p* = 0.062		
Over 4 chemotherapy cycles	9 (33.3%)	10 (37.0%)	*χ* ^2^ = 0.081		
			*p* = 0.776		
Radiation in hospital conditions	5 (18.5%)	10 (37.0%)	*χ* ^2^ = 2.308		
			*p* = 0.129		
Intrauterine probe ≥5 cm	20 (74.1%)	22 (81.5%)	*χ* ^2^ = 0.429		
			*p* = 0.513		
Rectal dose	62.089 (±11.184)	55.370 (±16.559)	*t* = −1.474		
			*p* = 0.087^a^		
Bladder dose	59.952% (±16.384)	52.223% (±13.136)	*t* = −1.910		
			*p* = 0.062^a^		
Total radiotherapy duration over 56 days	21 (77.8%)	17 (63.0%)	*χ* ^2^ = 1.421		
			*p* = 0.233		
Pause over 7 days	14 (51.9%)	5 (18.5%)	*χ* ^2^ = 6.577	6.384 (0.544–74.991)	*p* = 0.140
			*p* = 0.010		
Adherence to the proposed diet					
Yes	7 (25.9%)	11 (40.6%)	*χ* ^2^ = 2.222		
No	12 (44.4%)	12 (44.4%)	*p* = 0.329		
Partially	8 (29.6%)	4 (14.8%)			
Bladder volume (cm^3^)	273.653 (±41.652)	226.653 (±35.209)	*t* = −4.483	1.034 (1.003–1.085)	*p* = 0.034
			*p* < 0.001^a^		
Rectal volume (cm^3^)	133.596 (±24.649)	125.474 (±19.311)	*t* = −1.348		
			*p* = 184^a^		
Bowel bag volume (cm^3^)	792.690 (±85.687)	764.777 (±143.053)	*t* = −0.870		
			*p* < 0.388^a^		
Height ≥175 cm	9 (33.3%)	5 (18.5%)	*χ* ^2^ = 1.543		
			*p* = 0.214		
Weight ≤60 kg	12 (44.4%)	4 (14.8%)	*χ* ^2^ = 5.684	1.767 (0.047–66.503)	*p* = 0.758
			*p* = 0.017		
Body mass index (BMI) under 21 kg/m^2^	13 (48.1%)	21 (77.8%)	*χ* ^2^ = 5.082	0.745 (0.494–1.125)	*p* = 0.162
			*p* = 0.024		
Pelvic gross volume (cm^3^)	4675.74 (±934.061)	6162.667 (±1433.513)	*t* = 4.516	1.000 (0.998–1.003)	*p* = 0.276
			*p* < 0.001^a^		
Volume of the pelvic subcutaneous fatty tissue (cm^3^)	1384.180 (±0.555)	2402.629 (±0.959)	*t* = 4.772	0.998 (0.995–1.001)	*p* = 0.234
			*p* < 0.001^a^		

Data represent the mean value ± 1 standard deviation (SD).

^a^Student’s *t* test for independent samples.

We analyzed variables that had been proven to be statistically significant (*p* < 0.05) in the univariate analysis (Table 1). By using multivariate logistic regression, ACE inhibitors (OR_adjusted_ = 0.060, 95% CI = 0.004–0.817; *p* = 0.035) and bladder volume (OR_adjusted_ = 1.034, 95% CI = 1.003–1.085; *p* = 0.034) were isolated as factors with a potential impact on the ART. Hosmer-Lemeshow Goodness-of-Fit Test for logistic regression was Chi-square = 5.694; df = 8; *p* = 0.682 (Cox & Snell *R* ^2^ 0.605, Nagelkerke *R* ^2^ 0.807). There were significant variables after corrections for multiple comparisons (Table 3).

### 3.2 2D-RT

The main characteristics of the patients treated with 2D-RT, as well as the differences between groups, are presented in Table 2. Univariate analysis showed that all the following factors significantly affected ART occurrence: level of education and FIGO stage and specific drugs used during radiotherapy, the chemotherapy regime and the radiotherapy technique, ACE inhibitors and β-blockers therapy, more than four cycles of chemotherapy administration, treatment duration and treatment pause and parameters related to the brachytherapy application, such as intrauterine probe >5 cm in length, rectal dose and bladder dose. Among the analyzed patient’s constitutional characteristics, it was found that weight less than 60 kg, BMI, waist circumference and pelvic gross volume are significant factors in the occurrence of ART.

**Table 2:** Analysis of potential risk factors important for ART onset in patient treated with 2D-RT

Variables	Univariate analysis			Multivariate analysis	
	With toxicity (*n* = 42)	Without toxicity (*n* = 42)	Test and *p*-values	Exp(B) (95% CI for ExpB)	*p*
Age	56.07 (±11.221)	52.29 (±9.470)	*t* = 1.733		
			*p* = 0.087^a^		
Education groups of patients (years)					
Primary school	16 (38.1%)	5 (11.9%)	*χ* ^2^ = 7.839	0.310 (0.068–1.410)	*p* = 0.130
High school	20 (47.6%)	30 (71.4%)	*p* = 0.020		
College	6 (14.3%)	7 (8.3%)			
Charlson’s comorbidity-age combined risk score					
Score 2 (mildly ill)	7 (16.75%)	10 (23.8%)	*χ* ^2^ = 0.840		
Score 3 (moderately ill)	10 (23.8%)	10 (23.8%)	*p* = 0.840		
Score 4 (severely ill)	9 (21.4%)	9 (21.4%)			
Score 5 (severely ill)	16 (38.1%)	13(31.0%)			
Hypertension	13 (31.0%)	10 (23.8%)	*χ* ^2^ = 0.539		
			*p* = 0.463		
Smoking	28 (66.7%)	22 (52.4%)	*χ* ^2^ = 1.779		
			*p* = 0.182		
Alcohol	6 (14.3%)	3 (7.1%)	*χ* ^2^ = 1.120		
			*p* = 0.220		
Surgery in the abdomen or small pelvis	18 (42.9%)	10 (23.8%)	*χ* ^2^ = 3.429		
			*p* = 0.064		
Surgery before 10 years	10 (83.3%)	13 (61.9%)	*χ* ^2^ = 1.660		
			*p* = 0.198		
Patohistological type of tumor					
Squamocellular	33 (78.6%)	38 (90.5%)	*χ* ^2^ = 2.275		
Adenocarcinoma	9 (21.4%)	4 (9.5%)	*p* = 0.131		
FIGO stage					
IIb	11 (26.2%)	23 (54.8%)	*χ* ^2^ = 9.275	1.537 (0.676–3.494)	*p* = 0.305
IIIa	13 (31.0%)	12 (28.6%)	*p* = 0.026		
IIIb	12 (28.6%)	4 (9.5%)			
IVa	6 (14.3%)	3 (7.1%)			
ACE inhibitors	16 (38.1%)	6 (14.3%)	*χ* ^2^ = 6.158	0.037 (0.002–0.768)	*p* = 0.033
			*p* = 0.013		
β-Blockers	11 (26.2%)	4 (9.5%)	*χ* ^2^ = 3.977	5.513 (0.243–125.332)	*p* = 0.284
			*p* = 0.046		
Single dose of cisplatin	70.544 (±5.765)	72.349 (±6.496)	*t* = −1.347		
			*p* = 0.182^a^		
Over 4 chemotherapy cycles	13 (31.0%)	23 (54.8%)	*χ* ^2^ = 4.861	3.427 (0.574–20.473)	*p* = 0.177
			*p* = 0.027		
Radiation in hospital conditions	12 (14.3%)	7 (8.3%)	*χ* ^2^ = 1.700		
			*p* = 0.192		
Surface of radiation field (cm^2^)	279.909 (±50.467)	275.909 (±43.345)	*t* = 0.390		
			*p* = 0.289^a^		
Paraaortic field	5 (11.9%)	3 (7.1%)	*χ* ^2^ = 0.553		
			*p* = 0.457		
Energy (MV)	6.95 (±1.724)	7.33 (±1.908)	*t* = −0.960		
			*p* = 0.059^a^		
Intrauterine probe ≥5 cm	41 (97.6%)	35 (83.3%)	*χ* ^2^ = 4.974	53.092 (0.422–6683.327)	*p* = 0.107
			*p* = 0.026		
Rectal dose	64.605 (±15.178)	48.819 (±14.567)	*t* = 4.997	1.065 (1.001–1.133)	*p* = 0.045
			*p* < 0.001^a^		
Bladder dose	61.693 (±16.433)	43.282 (±13.621)	*t* = 5.590	1.072 (1.002–1.147)	*p* = 0.044
			*p* < 0.001^a^		
Total radiotherapy duration (days)	79.67 (±22.168)	64.05 (±25.251)	*Z* = −3.801	0.981 (0.926–1.038)	*p* = 0.506
			*p* < 0.001^b^		
Pause (days)	20.33 (±22.139)	6.79 (±12.193)	*t* = 3.474	1.075 (0.982–1.178)	*p* = 0.117
			*p* < 0.001^a^		
Adherence to the proposed diet					
Yes	12 (28.6%)	20 (47.6%)	*χ* ^2^ = 3.244		
No	20 (47.6%)	15 (35.7%)	*p* = 0.198		
Partially	10 (23.8%)	7 (16.7%)			
Height ≥ 175 cm	10 (23.8%)	6 (14.3%)	*χ* ^2^ = 1.235		
			*p* = 0.266		
Weight ≤ 60 kg	15 (35.7%)	5 (11.9%)	*χ* ^2^ = 6.563	4.854 (0.484–48.715)	*p* = 0.179
			*p* = 0.010		
BMI (kg/m^2^)	24.000 (±3.438)	26.000 (±4.710)	*t* = −2.223	0.709 (0.523–0.962)	*p* = 0.027
			*p* = 0.029		
Waist size	82.14 (±10.873)	86.67 (±9.869)	*t* = −1.997	1.063 (0.952–1.188)	*p* = 0.276
			*p* = 0.049^a^		
Bruto pelvic volume (cm^3^)	6753.34 (±1433.27)	7656.28 (±1480.94)	*t* = −2.839	1,000 (0.999–1.000)	*p* = 0.513
			*p* = 0.006^a^		

Data represent the mean value ± 1 standard deviation.

^a^Student’s *t*-test for independent samples.

^b^Mann–Whitney *U* test.

Using multivariate logistic regression, the following factors were identified, which significantly affect ART occurrence in patients treated with 2D-RT: ACE inhibitors (OR_adjusted_ = 0.037, 95% CI = 0.002–0.768; *p* = 0.033), BMI (OR_adjusted_ = 0.709, 95% CI = 0.523–0.962; *p* = 0.027), brachytherapy rectal (OR_adjusted_ = 1.065, 95% CI = 1.001–1.133; *p* = 0.045) and bladder dose (OR_adjusted_ = 1.072, 95% CI = 1.002–1.147; *p* = 0.044). Hosmer-Lemeshow Goodness-of-Fit Test for logistic regression was Chi-square = 3.118; df = 8; *p* = 0.927 (Cox & Snell *R* ^2^ 0.525, Nagelkerke *R* ^2^ 0.700). There were significant variables after corrections for multiple comparisons (Table 3).

**Table 3:** Benjamini–Hochberg correction for adjusted *p*-value

Variables	Unadjusted *p*-values	Adjusted *p*-values
**3D-CRT^a^**		
Smoking	0.028	0.028
ACE inhibitors	0.027	0.028
Pause over 7 days	0.010	0.020
Bladder volume (cm^3^)	0.000	0.000
Weight ≤ 60 kg	0.017	0.027
BMI less than 21 kg/m^2^	0.024	0.028
Pelvic gross volume (cm^3^)	0.000	0.000
**2D-RT^b^**		
ACE inhibitors	0.033	0.045
Rectal dose	0.045	0.045
Bladder dose	0.044	0.045
BMI (kg/m^2^)	0.027	0.045

^a^3D-CRT.

^b^2D-RT.

**Figure 1:**
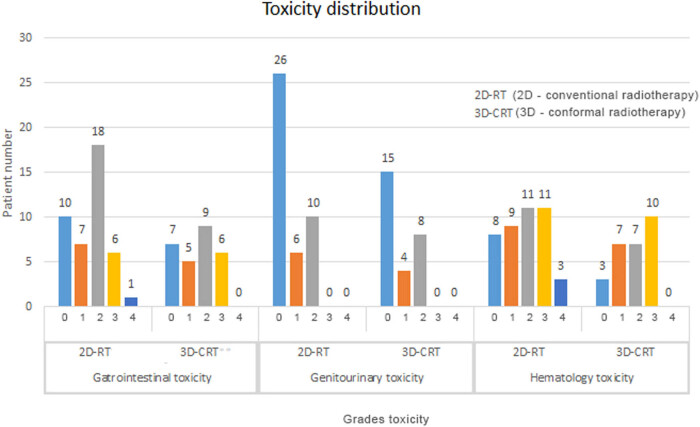
Distribution of acute radiation toxicity depending on radiotherapy technique.

The toxicity observed during the treatment was usually of mild to moderate intensity (Figure 1). In the group treated with 3D-CRT, there were no manifestations of ART of grade 4.

## 4 Discussion

The risk factors for ART occurrence can be identified (at a certain level) based on the anamnestic data and the data from the disease history and then by physical examination or simple anthropometric measurements.

It is interesting to note that brachytherapy dose escalation-associated factors have a significant effect on ART onset, while the brachytherapy initiation coincides with the peak of ART manifestation, especially in the case of genitourinary toxicity [7,8,27]. As with other studies, doses received via the rectum and bladder are factors of major influence. The ideal duration of the CCRT treatment should be between 50 and 55 days due to optimal compliance and treatment tolerance [28,29]. In developing countries, usually due to delays in intracavitary brachytherapy initiation, the treatment lasts for about 10 weeks on average [30]. In this study, it was shown that the treatment duration and the occurrence of pauses during radiotherapy, as well as the FIGO stage of the disease, significantly affected the onset of ART in patients treated with 2D-RT. For the majority of patients, prolonged treatment and the duration of treatment pauses were the result of technical and financial limitations, rather than the intensity and appearance of ART. In contrast, patients treated with 3D-CRT for the total duration of their radiotherapy showed no statistical significance for the onset of ART related to the aforementioned factors. Since most patients had mild to moderate radiation toxicity, they were paused during treatment for up to 7 days. However, only patients with delaying in initiating brachytherapy for technical reasons had pause over 7 days. Pause of over 7 days is still an important factor in the emergence of ART, unless it is a consequence of ART.

A very important aspect of each specific oncological therapy is the implementation of the total planned course of treatment. The literature suggests that only 65–92% of patients receive the planned number of chemotherapy cycles [31]. This is often caused by the appearance of serious manifestations of ART [31]. It has been shown that patients from the control group received more than four cycles of chemotherapy. This is statistically significant and suggests that they were in better general condition and thus able to endure a higher number of chemotherapy cycles. The appearance of ART disrupted the planned chemotherapy regime and in this way had a long-term effect on disease control and patient survival.

A recent study by Diaz et al. [32] confirmed that the presence of comorbidity is a very important prognostic factor in patients with cervical cancer. In earlier studies, the influence of patient personal therapy, applied simultaneously with radiotherapy, has been neglected in connection with the occurrence of ART. It is interesting that widely used ACE inhibitors are recognized as mitigators or as agents that can prevent the onset and decrease the intensity of ART. Their effect is most pronounced during and immediately after radiotherapy [33,34]. The Federal Drug Administration has confirmed that the treatment of hypertension with drugs in this group, in therapeutic doses, during a specific oncological treatment also reduces the growth of different types of tumors, which multiplies the effectiveness of the treatment several times [34]. It is known that ACE inhibitors during radiotherapy reduce the occurrence of radiation pneumonitis, nephropathy and optic neuropathy [29,35,36]. However, a detailed analysis of the literature found only one study conducted by Wedlake et al., concerning the analysis of the effect of ACE inhibitors on ART during radiotherapy of pelvic tumors [19]. They point out that ACE inhibitors, simultaneously with the use of statins, reduce gastrointestinal toxicity.

In this study, ACE inhibitors and β-blockers were found to have an effect on ART although the presence of hypertension was not shown to be a statistically significant factor (Tables 1 and 2). The case group patients had associated comorbidities that required a greater number of ACE inhibitors or beta blockers, in contrast to the control group patients. Although ACE inhibitors were identified as independent protective factors against the emergence of ART in both radiotherapy techniques, this study also raised suspicion that β-blockers might be involved because although multivariate analysis ruled out their influence, univariate was positive; with an increase in the sample size, the association of β-blockers and the ART may turned to be significant even after adjustment for other confounders. Beta-blockers may be useful in reducing radiation damage through modulation of the inflammatory response, but their importance should be further examined [11,12]. The results in Tables 1 and 2 indicate the control patients in both cohorts were stout, with a higher BMI and a higher volume of subcutaneous adipose tissue (Tables 1), indicating that they probably had a larger volume of abdominal fat. The literature data show adipocytes secrete hormones, growth factors and cytokines, the adipose tissue being an extremely hormonally active tissue. It was also found that angiotensin II, the major bioactive peptide hormone of the renin-angitensin-aldosterone system (RAAS), stimulates inflammation in adipocytes, potentiating in turn the formation of reactive oxygen species (ROS) via angiotensin type receptors 1 (AT1R) and NADPH oxidase interactions, followed by activation of MAPK/PI signaling pathways. Further, hypoxia, excessive ROS production, and oxidative stress can cause cell damage or death. When obesity is present, adipocytes secrete large amounts of angiotensinogens. Angiotensinogen mRNA expression is more pronounced in visceral than in subcutaneous fat. Inhibition of ACE, i.e. the blockade of AT1R and AT2R can be an interesting target for radioprotective therapy since it reduces oxidative stress and production of free radicals, which may indicate a protective anti-inflammatory effect in obesity, hypertension and other diseases, and reduction of damage caused by radiotherapy. There are data indicating that a particularly beneficial effect of antihypertensives affecting the RAAS system was observed in obese patients [37–39]. It might be that the protective effect of ACE inhibitors, determined by the statistical analysis of our study, was identified due to higher BMI of control patients in both cohorts, in whom a stronger mitigating effect of ACE inhibitors was shown when compared to the case group.

Other factors that were associated with the ART in the univariate analysis, but ruled out by the multivariate analysis, are weight, waist volume and pelvic gross volume. The possibility of their relationship with the ART merits new investigations with larger patient sample to differentiate the effects of confounders from true effects of these factors. The development of ART may be explained also by the patient’s constitution, regardless of the technique used. A study conducted by Smits et al. shows that obesity and BMI over 30 kg/m^2^ are not associated with higher grades of ART [40]. Similarly, another study show that obesity in young patients with endometrial cancer is not associated with the onset of genitourinary and gastrointestinal toxicities [41]. Lim et al. have found that obesity reduces the rectal dose during HDR brachytherapy due to larger quantity of fat tissue in the recto-uterine space, but does not affect the occurrence of acute gastrointestinal toxicity [42]. We have identified the following constitutional characteristics as predictive in the onset of ART: body weight less than 60 kg, small waist volume, low BMI and small pelvic gross volume. These results are confirmed by the analysis of the acute effects of 3D-CRT, in which weight ≤ 60 kg, BMI less than 21 kg/m^2^, pelvic gross volume, pelvic subcutaneous fatty tissue and bladder volume were identified as the most significant risk factors. This could be explained by the fact that the pelvic organs are concentrated in a small space. It is likely that the small intestines are fixed and positioned lower in the pelvis in patients with smaller body weight, without much fat tissue between, which implies a higher total volume of the intestines within the radiotherapy field. These results support the conclusion that the outcome of treatment is affected not only by the amount of fat tissue between the bowels but also by the volume of the pelvic subcutaneous fatty tissue. The occurrence of ART may also be affected by the volume of the bladder, as organ at risk.

The main factors that limit this study are the small sample size and unicenterdness as impact of local practices on outcome could not be excluded.

## 5 Conclusion

This study has shown that BMI, radiation dose received by the bladder and rectum are of exceptional importance for occurrence of the ART and also shown that therapy with ACE inhibitors was associated with the decreased chances of the ART. Since these drugs are often applied in practice, it is necessary to further examine their putative radioprotective effect for this indication.
